# Supplementary material for: The Mental Health Benefits of Purposeful Activities in Public Green Spaces in Urban and Semi-Urban Neighbourhoods: A Mixed-Methods Pilot and Proof of Concept Study
Source: Int J Environ Res Public Health. 2019 Jul 30;16(15):2712. doi: 10.3390/ijerph16152712 (PMC6696054; doi:10.3390/ijerph16152712)
Supplement: Supplementary file 1 [file ijerph-16-02712-s001.zip › Supplementary material 1.docx]

| **Individual context** | |
| --- | --- |
| *All participants green space and health context* | |
| 1 | What does green space mean to you? |
| 2 | What kind of green spaces do you like? |
| 3 | How healthy do you feel? |
| *Provider context* | |
| 4 | What are the motivating factors in the programme of practical conservation activity that you run. (PROMPT Is health and wellbeing a motivating factor? Where does this sit in the current priorities?) |
| 5 | Practical conservation is an existing part of your activity, but how would you see citizen science sitting with your aims as a programme? |
| 6 | How useful might citizen science data be to you and your organisation? |
| *Participant context* | |
| 7 | What first made you decide to attend this group? (PROMPTS people, spending time outdoors, health and wellbeing, activity and relative balance of factors) |
| 8 | Have your motivations changed over time? (PROMPTS people, spending time outdoors, health and wellbeing, activity and relative balance of factors) |

| **Context that day (for those who did more than one activity, repeat the Qus 9 to 12 as necessary)** | |
| --- | --- |
| 9 | How were you feeling generally on the day that you completed the activity? (PROMPTS for those who did more than one activity will need to remind them of which days they did each activity, was there anything happening that might have affected your mood) |
| 10 | How did you find filling the questionnaires before doing the activity? |
| *For those who did walking or citi sci* | |
| 11 | Were you expecting to do another kind of activity else that day? (PROMPT, if so, how did that make you feel?) |
| *For those who did practical conservation* | |
| 12 | How did you feel about doing your usual activity? (PROMPT do you like to do the same kind of thing, or do you like change? |
| *Qus relating to citizen science* | |
| 13 | How was the activity introduced? |
| 14 | What do you remember doing for the citizen science activity? |
| 15 | Were there particular things that you liked about doing the activity (PROMPTS looking in detail, new organisms, resources)? |
| 16 | Were there things that you didn't like about the citizen science activity? |
| *Qus relating to walking* | |
| 17 | Could you tell me about what you did and where you went on the walk? |
| 18 | Were there particular things you liked about going for a walk? (PROMPTS talking, exercise, novelty, purpose) |
| 19 | Were there particular things you didn't like about going for a walk? (PROMPTS talking, exercise, novelty, purpose) |
| *Qus relating to practical conservation* | |
| 20 | Could you tell me about the practical conservation activity that you did? |
| 21 | Were there particular things you liked about the activity? (PROMPTS talking, exercise, novelty, purpose) |
| 2 | Were there particular things you didn't like about the activity? (PROMPTS talking, exercise, novelty, purpose) |
| *Qu for participants who did more than one activity* | |
| 23 | Which activity did you like most? Why was that? |
| **Qus relating to the activity in general** | |
| 24 | What did you enjoy most about the activity? (PROMPTS talking, exercise, novelty, purpose) |
| **Qus relating to the nature of the space** | |
| 25 | Tell me about the site (PROMPTS is it somewhere you'd been before or go to regularly?) |
| 26 | Are there particular things that appeal to you about the site? (PROMPTS location, accessibility, species make up and habitats, sensory aspects) |
| 27 | Are there aspects that you don’t like? (PROMPTS location, accessibility, species make up and habitats, sensory aspects) |

| **Post activity questions** | |
| --- | --- |
| 28 | What do you remember most from the activity/ies? |
| 29 | Have you done any of the activities again since? |
| 30 | Would you do citizen science or going for a walk with a group again if there was an opportunity? |
| 31 | Would you do citizen science or go for a walk on your own? |
| **If we were to do this again….** | |
| 32 | If the activity you were asked to undertake was chosen at random on the day, would it make you less inclined to participate? |
| 33 | Is there anything else that you would suggest we do differently? |
